# Influence of solubilization and AD-mutations on stability and structure of human presenilins

**DOI:** 10.1038/s41598-017-18313-x

**Published:** 2017-12-21

**Authors:** Ge Yang, Kun Yu, Christina-Symina Kaitatzi, Abhilasha Singh, Jörg Labahn

**Affiliations:** 1 Centre for Structural Systems Biology (CSSB), CSSB-FZJ, Notkestr. 85, 22607 Hamburg, Germany; 2 Institute of Complex Systems-Structural Biochemistry (ICS-6), Forschungszentrum Jülich, Wilhelm-Johnen-Str., 52425 Jülich, Germany; 3 Physics Department, University of Patras, University Campus, 26504 Rio Achaia, Greece; 4 Institut für Physikalische Biologie, Heinrich-Heine-University Düsseldorf, Universitätsstraße 1, 40225 Düsseldorf, Germany

## Abstract

Presenilin (PS1 or PS2) functions as the catalytic subunit of γ-secretase, which produces the toxic amyloid beta peptides in Alzheimer’s disease (AD). The dependence of folding and structural stability of PSs on the lipophilic environment and mutation were investigated by far UV CD spectroscopy. The secondary structure content and stability of PS2 depended on the lipophilic environment. PS2 undergoes a temperature-dependent structural transition from α-helical to β-structure at 331 K. The restructured protein formed structures which tested positive in spectroscopic amyloid fibrils assays. The AD mutant PS1L266F, PS1L424V and PS1ΔE9 displayed reduced stability which supports a proposed ‘loss of function’ mechanism of AD based on protein instability. The exon 9 coded sequence in the inhibitory loop of the zymogen was found to be required for the modulation of the thermal stability of PS1 by the lipophilic environment.

## Introduction

Human presenilins (PS1 and PS2) are involved in many physiological and pathological events, e.g. Alzheimer’s disease (AD), by cleaving over 90 different substrates^1–3^. PSs are membrane proteins with 9 transmembrane segments (TM). They are homologs with 67% sequence identity^4^ and belong to the peptidase A22A family and the ER calcium leak channel presenilin family^5^. The proteolytic activity of PSs requires the proteins nicastrin, APH-1 and PEN-2 to form the γ-secretase complex and activation by cleavage of PSs^6–8^. Full length PSs were suggested to be an ER calcium leakage channel^5^, though the ER pool of PS2 was found to be low^9^.

For proteolytic activity PS zymogens undergo activation by auto-cleavage in the α-helical region of the third cytoplasmic loop between TM6 and TM7. It was proposed that auto-cleavage induces changes in conformation resulting in either release of the inhibitory exon 9 loop from the putative substrate binding site^10^ or the rearrangement of the catalytic aspartyl dyad required for cleavage^11^.

In γ-secretase the PSs transmembrane helices, especially TM2, are loosely packed^12^. γ-secretase also adopts different conformations with varied compactness of the complex and changes of the relative position of the nicastrin ectodomain, while familial AD (FAD) related mutations in PS result in enrichment of structures with increased flexibility^13^. The physical and pathological importance of the structural flexibility is poorly understood.

γ-secretase processes the amyloid precursor proteins (APP-C99) to generate amyloid beta (Aβ) peptides of various length^1^ that organize into Aβ fibrils^14^. Changes in the lipid microenvironment (e.g. fatty acid chain length) were reported to play an important role in regulating the substrate processing of γ-secretase and the ratio of the generated non-toxic and toxic Aβ peptides^15^. The actual mechanism of AD pathogenesis is still under debate. According to the gain of function hypothesis mutations in PSs alter the cleavage pattern and produce longer, aggregation prone Aβ peptides which are considered to be the cause of AD^16^. However, loss of function of PS in γ-secretase could better explain the cause for AD given that some AD mutations reduce the generation of toxic Aβ, which may implicate the calcium leakage channel function^5,17,18^. Recently, a simulation study based on full length PS proposed that mutations cause AD via influencing the overall structural stability of PS. This could explain why mutations scattered across the protein could alter the cleavage pattern of the substrate^19^.

We prepared complexes of human full length PSs and AD related mutants with lipid-like fos-choline detergents based on the results of the presenilin expression study^20^ to investigate the effect of the lipophilic environment on folding and stability of the proteins, because our previous finding that presenilin 2 exhibited a low melting temperature for tertiary structure (34 °C), but relatively stable secondary structure (Tm 55 °C) pointed at thermally induced refolding. We report here the influence of solubilization and mutation on the structure and the thermal stability of PSs.

## Results

### Effect of detergent on the purification and oligomerization of presenilins

We purified PS2 using fos-choline detergent with chain length of 12 (FC12), 14 (FC14) and 16 (FC16) to investigate the effect of detergent chain length on the purification yield and biophysical property of the PS2-detergent complex. The critical micelle concentrations of the used detergents were determined for the used buffers (see Supplementary Figure S11).

**Figure 1:**
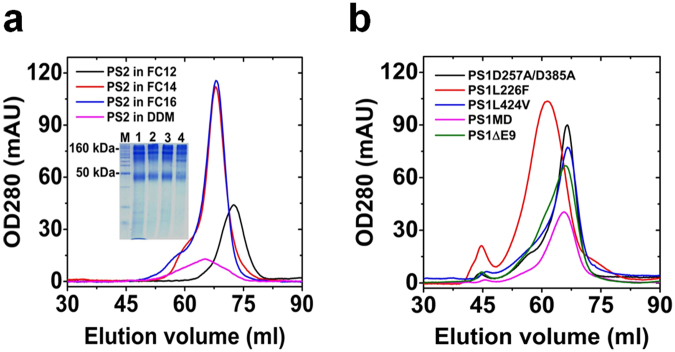
Detergent dependence of oligomerisation for presenilins. Analysis of apparent size of (**a**) PS2 in different detergents and (**b**) PS1 mutants in FC16 by SEC. Inset in (**a**) blue-silver stained 15% SDS-PAGE of purified PS2. Lane 1: FC12 purified PS2; lane 2: FC14 purified PS2; lane 3: FC16 purified PS2; lane 4: DDM purified PS2; M: BenchMark protein ladder (Invitrogen). See Supplementary Figure S1 for first SEC purification. For data on column calibration see Supplementary Figure S2. For estimation of the oligomerization state see Supplementary Table S1.

The yield after Ni-NTA was similar for FC12, FC14 and FC16 as the total peak areas were identical. However, the yield for DDM was not only significantly lower but also showed almost no un-aggregated material. We were unable to produce reasonable amounts of PS2 in DDM of sufficient quality for further biophysical investigation. For FC12, four apparent un-separated peaks were seen on SEC, whereas for FC14 and FC16 only two major peaks were observed (see Supplementary Figure S1). Samples with increased homogeneity for biophysical characterization were obtained by a second SEC^20^ from the pooled fractions (Fig. 1).

The purified homogeneous PS2 pools (Fig. 1a) exhibited before and after correction for the detergent micelle different apparent molecular weights in different detergents (see Supplementary Figure S2 and Table S1). In FC12 and FC16 PS2 existed clearly as a dimer, while PS2 in FC14 was probably mainly in trimeric form. PS1 exhibited similar apparent oligomerization states as PS2 though in FC14 the dimeric oligomerization state appears to be preferred with respect to PS2. The functional mutation PS1D257A/D385A and the FAD mutation PS1L424V showed identical behavior as the native protein. For the mutations PS1ΔE9 and PS1M292D, a slight increase towards trimerisation was calculated. PS1L226F which is the most potent FAD mutation investigated, however showed a significantly stronger oligomerization than all other PSs (Fig. 1b and also see Supplementary Figure S1 and Table S1).

### Effect of detergent on the secondary and tertiary structure of presenilins

**Figure 2:**
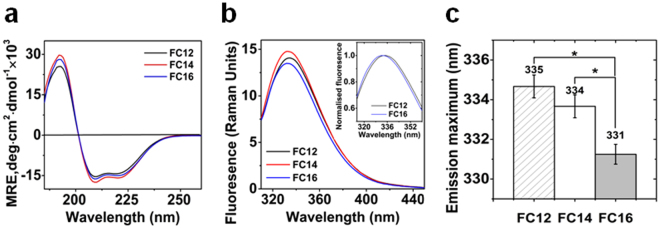
Effect of detergent on the secondary and tertiary structure of presenilins. (**a**) Far UV CD spectra of PS2 purified in different detergents. (**b**) Tryptophan fluorescence emission spectra of PS2 in different detergents; inset: overlay of fluorescence emission spectra in FC12 and FC16. (**c**) Emission maxima of PS2 tryptophan fluorescence in different detergents; the differences between group means are analyzed using the one-way ANOVA test (n = 3) at significance level of 0.05; *Represents that the difference is significant (P < 0.05).

Presenilins purified with detergents of different chain length showed different far UV CD (circular dichroism) spectra with varied intensity (Fig. 2a and see Supplementary Figure S3). The deconvolution showed that FC14 purified PS2 had the highest helical and least strand content (prediction based on the incomplete EM structure of PS1 in γ-secretase^12^ by RaptorX^21^: 53% helix and 8% strand) (Table 1). A similar tendency was observed for PS1 too (see Supplementary Figure S3).

**Table 1:** Secondary structure content of presenilin 2.

Bestsel	Helix		Strand				Turn	Others	NRMSD
Sample	Helix1	Helix2	Anti1	Anti2	Anti3	Para			
PS2 in FC12	23.47	13.78	0.26	3.2	2.06	4.6	12.92	39.72	0.0032
PS2 in FC14	28.74	16.38	0	0.63	0	2.45	12.8	38.99	0.0035
PS2 in FC16	25.35	13.72	0	2.24	0	4.43	13.11	41.15	0.004

The effect of detergent on the tertiary structure of PS2 was probed by measurements of intrinsic tryptophan fluorescence (Fig. 2b). The fluorescence spectra showed different maximal intensity. FC14 exhibited the highest quantum yield. The emission maximum showed a blue shift as the chain length of the detergent increased (Fig. 2b inset). FC16 purified PS2 had an emission maximum of ~331 nm which was significantly different from the other two (Fig. 2c). These differences are caused by differences in interaction of tryptophan and detergent. The tryptophan residues of FC12 and FC14 purified PS2 are more exposed to the hydrophilic solvent environment than those of FC16 purified PS2.

### Effect of detergent on the thermal stability of presenilins

**Figure 3:**
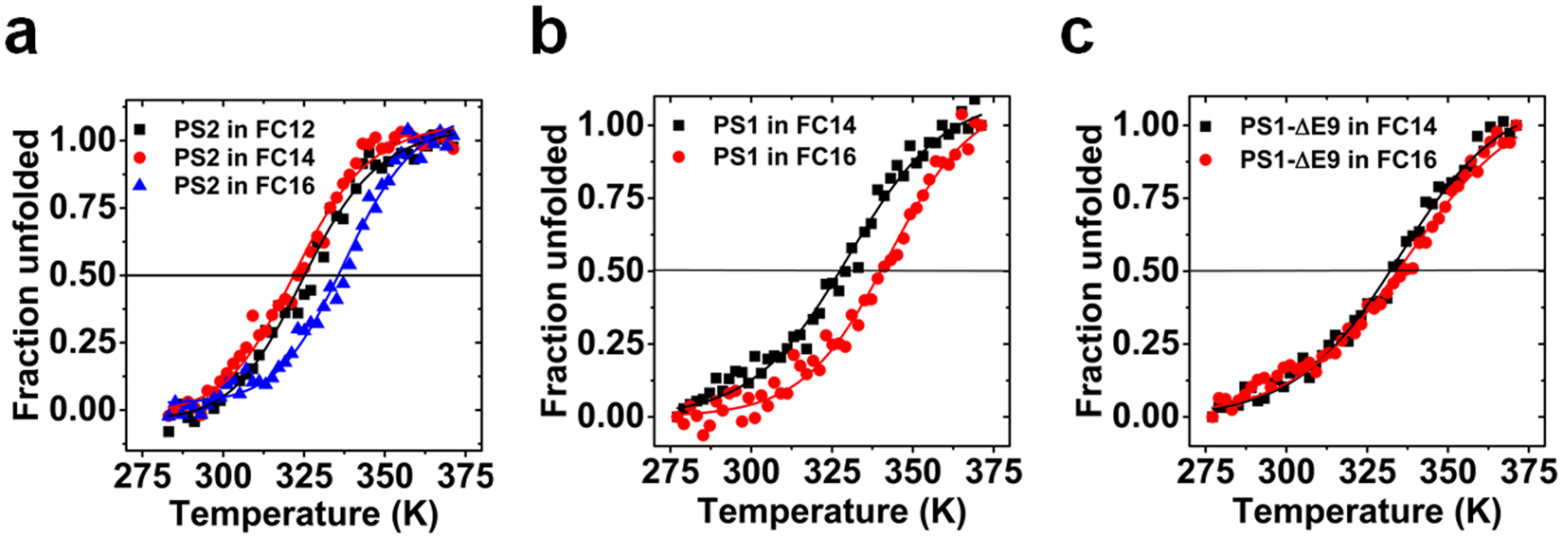
Effect of detergent on the thermal stability of presenilins. Fraction unfolded calculated by equation 1 (see Methods) from CD signal as a function of temperature. (**a**) PS2 in FC12, FC14 and FC16 (**b**) PS1 in FC14 and FC16 (**c**) PS1-ΔE9 in FC14 and FC16.

The dependence of stability on detergents was investigated by thermal unfolding of PS2 in lipid-like fos-choline detergents with varied chain length (Fig. 3a). Non-linear regression analysis using equation (2) (see Methods) showed different thermal stabilities for PS2 in response to detergent chain length. PS2 in FC12 and FC14 exhibited a similar T_m_ with 325.3 ± 0.9 K for FC12 and 322.9 ± 0.9 K for FC14. However, in FC16 the T_m_ was 338.4 ± 0.9 K, a substantial 15 K increase as compared with FC14. Increasing FC14 and FC16 concentrations (w/v) to the higher value required for of FC12 did not alter the transition temperatures of PS2 (see Supplementary Figure S12).

Similarly, PS1 also displayed different thermal stabilities with a T_m_ of 330.1 ± 0.5 K in FC14 and 342.5 ± 0.5 K in FC16 (Fig. 3b). However, thermal unfolding of PS1-ΔE9 in these two detergents showed, unlike the PS1WT, similar T_m_ values of 334.9 ± 0.4 K in FC14 and 337.6 ± 0.4 K in FC16 (Fig. 3c).

### Secondary structure transitions of presenilin2 in FC14

**Figure 4:**
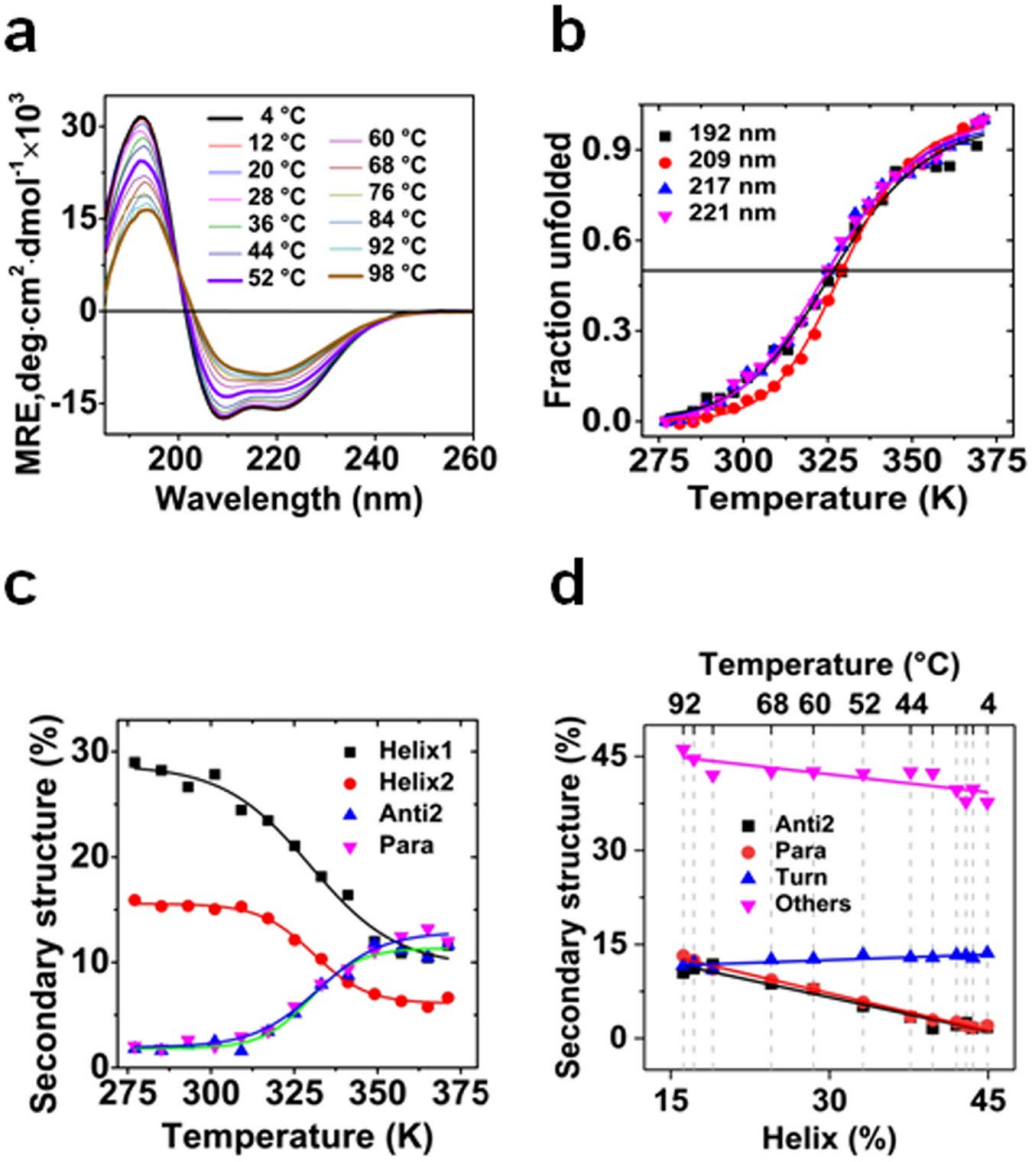
Thermal unfolding of presenilin2 in FC14 monitored by far-UV CD. (**a**) CD spectra (260 nm to 185 nm) shown in 8 °C increments from 4 to 98 °C; (**b**) Fraction unfolded calculated from signal at 192, 209, 217 and 221 nm respectively. Solid lines represent the fitting of the data into equation (2). (**c**) Helix and strand content at different temperatures after deconvolution. Solid lines represent the fitting of the data into equation (2) (see Methods); (**d**) Correlation of helical structure with other secondary structures for data points at different temperatures. Solid lines represent a linear fit. For details of data deconvolution see Supplementary Figure S5. For details on the control of protein precipitation during unfolding see Supplementary Figure S4.

CD spectra between 260 nm and 185 nm were collected at increasing temperatures to investigate the structural transitions of PS2 in FC14 during thermal unfolding (Fig. 4a). The spectra showed as observed previously a typical spectrum for a helical protein with a double minimum at ~209 nm and ~221 nm as well as a positive peak at 192 nm at low temperatures^20^. The intensity of these three peaks progressively decreased as the temperature increased till 371.15 K, where the spectrum exhibited a single minimum at 217 nm characteristic for a beta-strand rich protein. The spectra showed an isodichroic point at 200 nm indicating the presence of a two-state transition. From the MRE (mean residue ellipticities) at 192, 209, 217 and 221 nm as a function of temperature the T_m_ values of 327.1 ± 1.2, 329.4 ± 0.5, 325.1 ± 0.8 and 325.6 ± 0.8 were obtained respectively (Fig. 4b).

The change of the absorption calculated from the dynode voltage at 221 nm indicated that there was no significant change of protein concentration during thermal unfolding (see Supplementary Figure S4). This allowed the deconvolution of the CD spectra at each temperature into component spectra (see Supplementary Figure S5), though it was previously shown that the unfolding of PS2 in FC14 was irreversible^20^. For temperatures lower than 300 K the total helical structure content remained at ~43% (Fig. 4c). Upon further temperature increase the signal decreased and reached a plateau at ~18% which corresponded to a loss of ~25% of the helical structure. The total β-strand content showed a mirrored transition with an initial content of 3.8% followed by an increase after 317.15 K reaching a plateau of ~26% (an increase of ~22%). The turn contents remained constant during thermal unfolding while other structural content increased slightly (see Supplementary Fig S5).

The algorithm BeStSel differentiated anti-parallel and parallel beta sheet and detected an increase of these contents during unfolding which is an indicator for aggregation. The analysis of the temperature dependence of regular helical structure content (helix1), distorted helical structure (helix2), relaxed antiparallel beta-strand (anti2) and parallel beta-strand (para) content showed a two state transition (Fig. 4c): Fitting the secondary structure content into a two-state model resulted in T_m_ values of 329.8 ± 2.0 K, 330.7 ± 0.9 K for helix1 and helix2, respectively and T_t_ values of 329.9 ± 1.7 K and 332.2 ± 1.4 K for the beta structures respectively, which is in good agreement with the transition temperatures obtained directly from the experimental data (s.a.).

The thermally induced structure transitions were strongly (linearly) correlated: A scatter plot of non-helical secondary structural content against the disappearing helical structural content showed the strong correlation of the individual structural transitions with slopes of −0.37 (r^2^ = 0.968), −0.40 (r^2^ = 0.993), 0.06 (r^2^ = 0.713) and −0.20 (r^2^ = 0.728) for anti2, para, turn and others respectively, (Fig. 4d), which indicated a coordinated structural transformation at about 331 K.

### Thermal unfolding of presenilin2 in FC14 monitored by thermal shift assay

**Figure 5:**
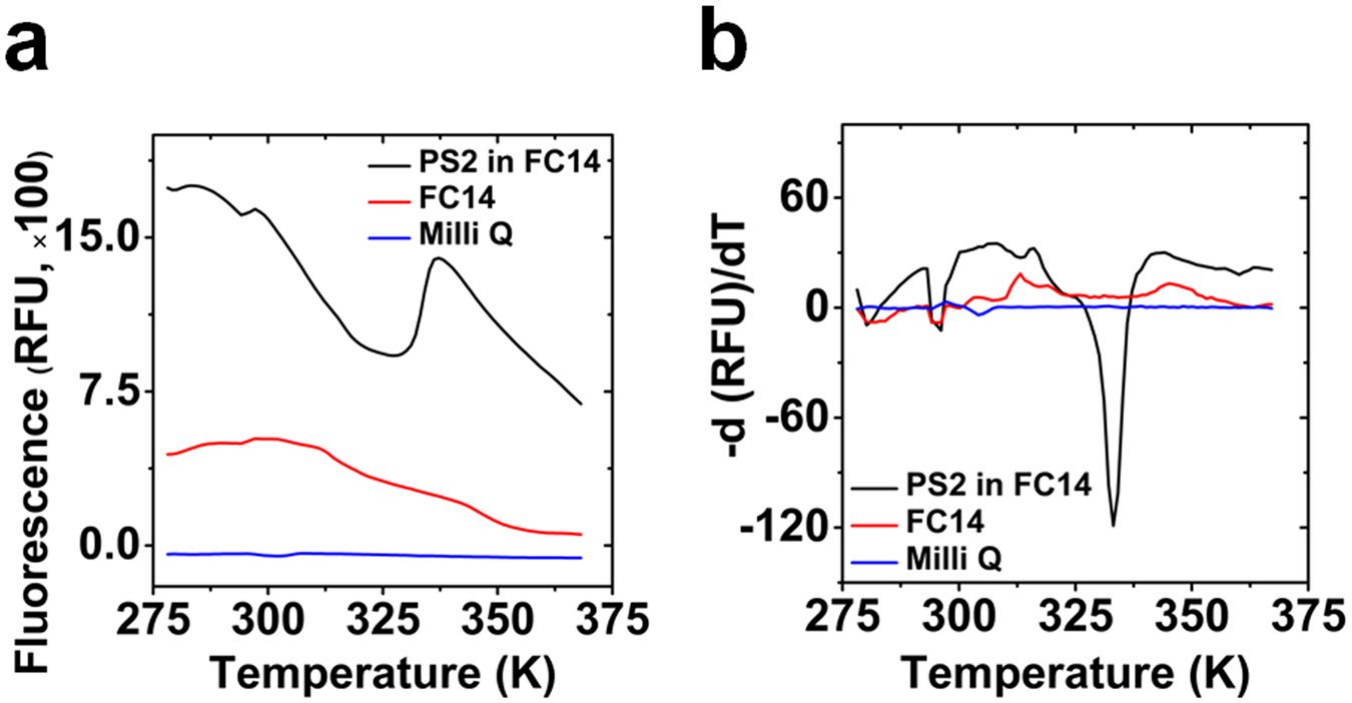
Thermal unfolding of presenilin2 in FC14 probed by fluorescence of hydrophobicity sensitive SYPRO Orange dye. (**a**) SYPRO orange fluorescence thermal unfolding curve as a function of temperature; (**b**) First derivative of the SYPRO orange fluorescence intensity as a function of temperature. Controls: buffer containing FC14 and Milli-Q water.

Thermal unfolding of PS2 in FC14 was also investigated using the dye SYPRO orange (SO), which is sensitive to changes in the hydrophobic environment, to access the change of exposed hydrophobic surface. Figure 5 shows the raw SO fluorescence signals (a) and the first derivatives (b) as a function of temperature. The signal intensity decreased almost linearly from 295 K to 328 K followed by a steep increase (max at 337 K). Thereafter the signal decreased linearly with similar slope as before the increase. The SO fluorescence signal exhibited these extrema with increasing temperature only in the presence of PS2. From the first derivative a transition temperature (T_m_) of 333.15 K was obtained. The same analysis for controls showed no significant minima.

### Temperature-dependent formation of SDS-resistant oligomers

**Figure 6:**
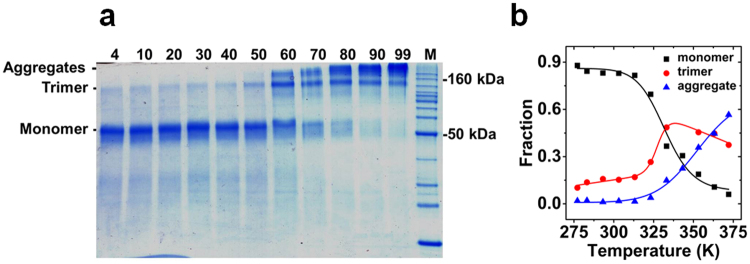
Temperature induced aggregation of presenilin2 visualized by SDS-PAGE. (**a**) The same concentration of PS2 in FC14 was heated at identical heating rate as in the CD melting experiment. At the respective temperatures (°C, top of lane) aliquots of 3.5 µg of PS2 were applied to a 12% SDS-PAGE; (**b**) Densitometric analysis of aggregation of PS2 on SDS-PAGE. Solid lines represent the fitting of the data into equation (2) (see Methods).

Thermal unfolding of membrane protein often resulted in the formation of SDS-resistant aggregates^22,23^ which might disturb measurements by e.g. precipitation. The aggregation behavior of PS2 in FC14 induced by thermal unfolding was studied by separation of the irreversibly formed aggregates and subsequent densitometric analysis by SDS-PAGE (Fig. 6a). From 277.15 K to 323.15 K PS2 existed mainly in monomeric form on SDS-PAGE. Upon temperature increase a loss of monomer and an increase of the SDS resistant trimer and high molecular weight aggregates were observed. Densitometric analysis (Fig. 6b) of the signal revealed a T_t_ of 332.1 ± 2.0 K for the loss of monomer and a T_t_ of 327.9 ± 0.8 K for the formation of SDS-resistant trimer. Therefore thermal restructuring of PS2 followed a two state transition which consequently lead to the formation of SDS resistant oligomers and aggregates. This aggregation disallows the analysis of the unfolding process by equilibrium thermodynamics^24^. Although thermal unfolding of PS2 is an irreversible process accompanied by aggregate formation, the apparent T_m_ could still be used to compare the stability of the protein-detergent complexes^25,26^ because the kinetics of aggregation did not disturb the unfolding (see Supplementary Figure S6): Similar T_m_ values were obtained with different heating rates.

### Spectroscopic analysis of aggregates

**Figure 7:**
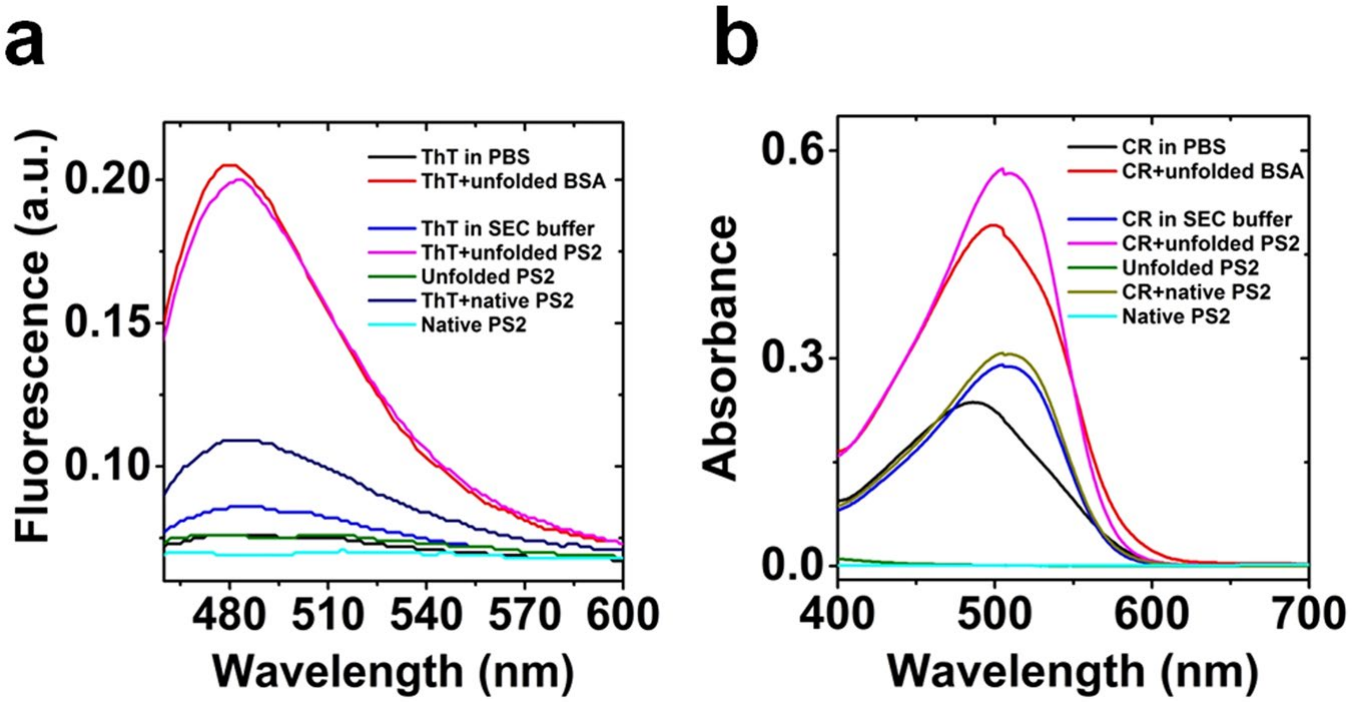
Fibrilisation assays for presenilin2 in FC14. (**a**) Thioflavin T binding assay: Fluorescence spectra of native and unfolded PS2 and positive control BSA with and without ThT; (**b**) Congo red absorption assay: Absorption spectra for native and unfolded PS2 and positive control BSA. For experimental details see Methods. See Supplementary Figure S7 for the far UV CD and absorption spectra of folded and unfolded protein.

During thermal unfolding beta-sheet rich aggregation was detected. We therefore studied whether PS2 formed aggregates positive in Thioflavin T (ThT) and Congo Red (CR) assays. In both assays PS2 showed binding to the amyloid fibril specific dyes ThT (Fig. 7a) and CR (Fig. 7b) similar to the positive control BSA which forms fibrils^27–29^. The protein free detergent control showed a marginal false positive signal due to the interaction of dyes with detergent. For CR the red shift upon binding detergent or unfolded protein was quite comparable though the change of absorption intensity clearly allowed differentiating between the detergent effect and aggregate labeling. For ThT however only the detergent effect on intensity was clearly detectable. Native protein displayed far UV CD spectra for helical protein, whereas for unfolded presenilin2, spectrum for beta-rich protein was observed. The absorption spectra between native and unfolded protein were similar (see Supplementary Figure S7).

Analysis of re-cooling experiments furthermore showed the structural transformation of secondary structure was partially reversible. Most noteworthy, the content of anti-parallel beta-structure formed upon heating showed no reversibility (see Supplementary Figure S8).

### Effect of AD related mutations on the secondary and tertiary structure and the thermal stability of presenilin1

**Figure 8:**
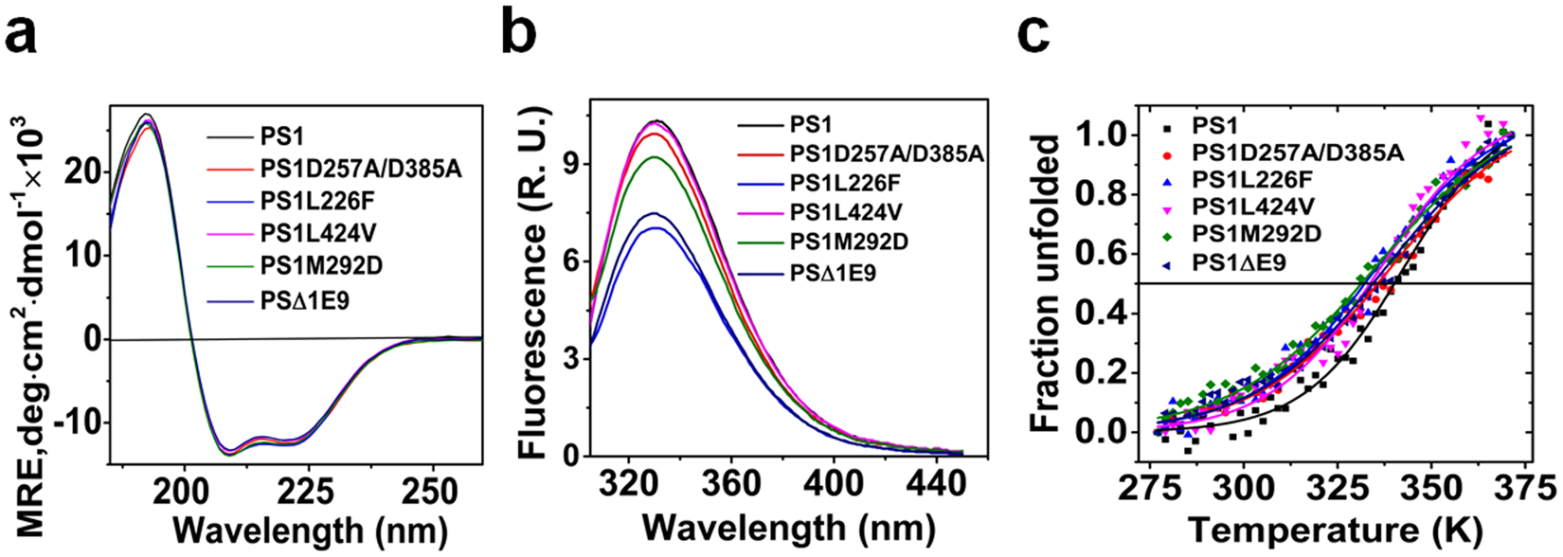
Far UV CD, fluorescence spectra and thermal stability of AD related mutations in presenilin1 in FC16. (**a**) Overlay of far UV CD spectra of PS1 AD mutants; (**b**) Tryptophan fluorescence emission spectra of PS1 AD mutants; (**c**) Thermal unfolding of PS1 AD mutants. For deconvolution of far UV CD spectra and list of melting temperatures see Supplementary Figure S9.

Because most AD mutations were found in PS1, we investigated PS1 AD mutations for stability. Five PS1 mutants were expressed and purified (see Methods). In detergent these mutants could be purified to homogeneity with various apparent molecular weights (see Fig. 1b and Table S1). The purified protein-detergent complexes were subjected to far and near UV CD and fluorescence spectroscopy to study the effect of mutation on the secondary and tertiary structure as well as the thermal stability of PS1 (Fig. 8). PS1 AD mutants displayed similar shape and intensity of far UV CD spectra with minor difference compared to the wild type (Fig. 8a). The helical structure content of the mutants was slightly lower than for PS1WT (see Supplementary Figure S9). However, the tryptophan fluorescence quantum yield varied for the different mutants although the emission maxima remained at ~331 nm (Fig. 8b) indicating different microenvironments of the tryptophan residues caused by different tertiary structures without significant changes of the interaction with the hydrophobic environment. Thermal unfolding of PS1 AD mutants showed varied melting curves (Fig. 8c) with lower T_m_ values (see Supplementary Figure S9) than the wild type protein. This is in accordance with the reduced stability predicted for point mutations based on the structure of wild type PS1.

**Figure 9:**
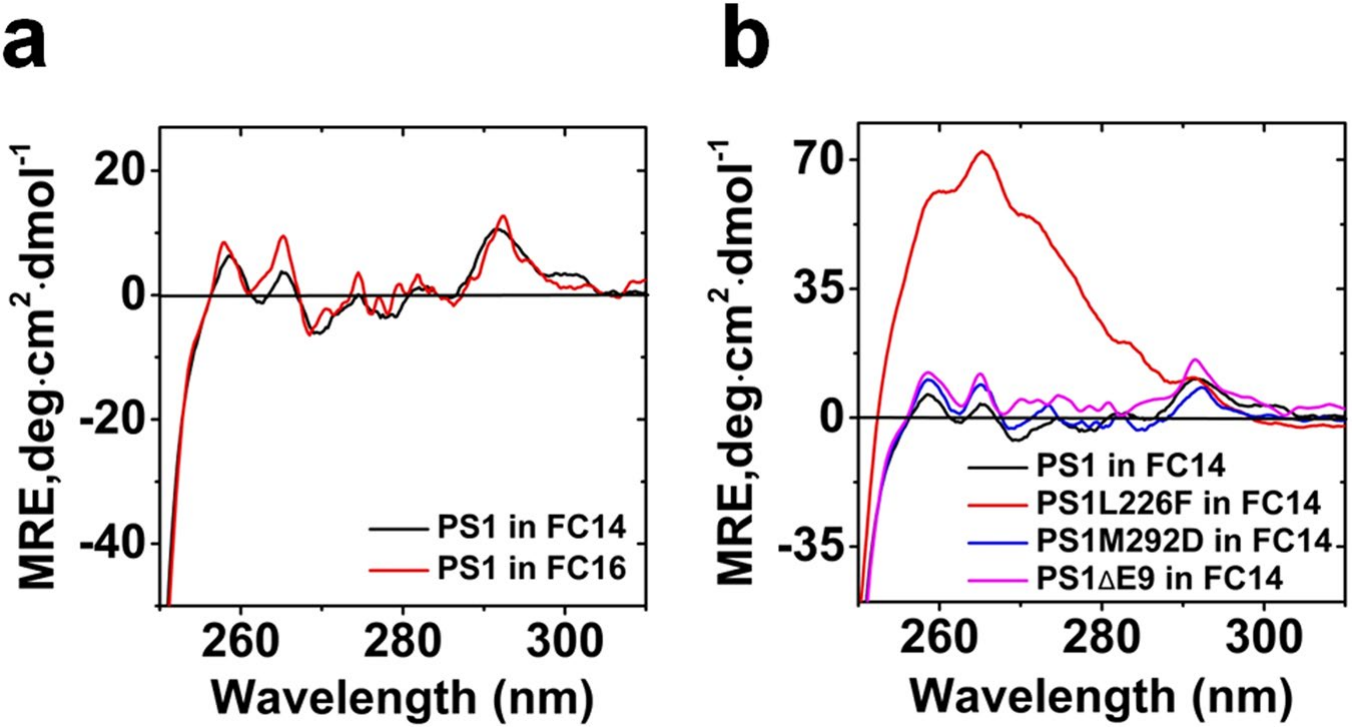
Near UV CD of AD related mutations of presenilin1. (**a**) Near UV CD spectra of PS1 in FC14 and FC16; (**b**) Near UV CD of PS1 mutations in FC14. See Methods for experimental parameters. Deconvolution of PS2 far UV CD spectra in different detergents using BeStSel 54 (see Methods). Structure content shown in percent.

Solubilization of PS1 by detergents of different chain length caused only small differences in near UV CD (Fig. 9a) and far UV CD spectra (see Supplementary Figure S3). The deconvolution however showed that the different detergents can already induce a small transition of helical to beta structure (see Supplementary Figure S3). When comparing the near UV CD spectra of several PS1 mutants, the signal for tryptophan at around 292 and for phenylalanine centred at 259 nm and 265 nm varied slightly to different extent except for PS1 L226F which showed almost ten times higher signal for phenylalanine (Fig. 9b). PS1 E9 shows differences in the near UV CD region at ~275 nm and at 292, which is in agreement with the differences observed for the intrinsic fluorescence (Fig. 8b).

## Discussion

Of all the AD pathogenic mutations, about 150 are mapped onto PS1, but only about 13 onto PS2)^30^. It was proposed based on simulations that the mutations change the overall stability of PSs which would explain why mutations far away from the catalytic sites could still influence the cleavage pattern and cause AD^19^. But hydrophobic interactions between the lipids and the membrane protein play also an important role in stabilizing membrane protein structures and functions. Therefore the hypothesis that AD mutations might cause the elevated production of toxic Aβ peptides because of decreased protein stability^19^ could be due to a decreased stability of the mutant or the unfavourable interaction of the mutant with their hydrophobic environments. Matching the hydrophobic surface of the membrane protein by the acyl chains of the lipids stabilizes and regulates the optimal activity of membrane proteins^31,32^ including presenilin^15^. This explains the importance of the choice of detergent in sample preparation: Improved thermal stability as the chain length increased was reported for e.g. rhodopsin^33^ and mitochondrial carriers^34^.

In this work, we observed that PSs exhibited decreased thermal stability as the chain length of lipid-like fos-choline detergents decreased, whereas excessive detergent concentrations did not change the stability. All three AD-mutants displayed also reduced stability in accordance with a ‘loss of function’ AD hypothesis^19^. Previous attempts trying to investigate the correlation of the structural stability of a PS1 fragment consisting of TM6 and TM7 with the AD pathogenicity found no relation between the AD mutations and stability^35^. In contrast, we found that all AD mutation of full length presenilin studied, displayed a decreased thermal stability as expected. Apparently the TM6/TM7 construct is too small to represent a suitable model for presenilin and therefore the ‘loss of function’ AD hypothesis is still permitted.

The point mutation PS1M292D and the deletion construct PS1ΔE9 which misses the exon 9 coded sequence are fully functional in amyloid-genesis without proteolytic activation^36,37^. For the mutant PS1M292D it had been suggested that the mutation affects the structure of the auto-inhibitory E9 loop and would cause similar behavior as the E9 deletion^11^. The exon 9 coded sequence was proposed to modulate substrate binding and enzyme activation^10,11^, and the potential calcium leakage channel function of PSs^38^. We showed here that the exon 9 coded sequence is required for the modulation of thermal stability by the lipophilic environment. But this structure element is cleaved in Aβ producing γ-secretase. Therefore it appears more likely that the suggested calcium leakage channel function is modulated by the lipidic environment.

The AD mutation PS1L226F, located in TM5, was reported to increase Aβ42 and Aβ40 production and Aβ42/Aβ40 ratio^39^. In a comparative study this mutant was found to display the largest increase of Aβ40 production and was the most active in Aβ42 production^40^. PS1L424V, located in TM8, exhibited similar affects in Aβ production, but displays the atypical phenotype of early onset AD^41^. We observed that PS1L266F exhibited the least helical content, fluorescence intensity, the highest near UV CD signal and thermal stability. In line with this observation, PS1L266F apparently exists in FC16 as a tetramer, which is different from the rest of the mutated proteins (see Supplementary Table S1). These results suggest that PS1L266F mutation alters the folding resulting in the reduced stability of PS1. The PS1L424V differed in oligomerization state, and the intrinsic fluorescence significantly from PS1L266F. This indicated substantial differences in tertiary structure, even though the secondary structures were similar.

CD is a powerful spectroscopic technique to investigate the type of structural transition during thermal unfolding of a protein^25^. The deconvoluted component spectra of presenilin showed a thermally inducible structural transition from helix to strand. Half of the helical content restructured. Whereas the content of unordered structure and turns increased only slightly, more than ~60% of helical structure turned into β-structure. The linear dependence and the presence of an isodichroic point are evidence for the existence of two different secondary structures of the protein: A low temperature form rich in α-helices and a high-temperature form rich in β-structures. This suggests that the oligomers and aggregates stabilize the refolded state. This is further supported by the irreversible formation of SDS-resistant oligomers and aggregates, which could be expected to accumulate in lysosomes^9^.

The thermal shift assay revealed a typical melting curve with a major transition starting around 327.15 K, the T_m_ of the secondary structure, which indicated that the transition was caused by protein unfolding. Upon structural transition, before the Tm was reached, the binding of SO increased, which indicated an increase of the exposed hydrophobic surface of the protein. Beyond the Tm the binding of SO decreased, which indicated a release of SO from the exposed hydrophobic surface upon beta-strand formation as revealed by CD. The transition temperatures calculated from the thermal shift assay (ca. 333 K) were in a reasonable agreement with the structural changes (330–333 K) from CD spectroscopy.

Similar transitions upon thermal unfolding of other proteins resulted in the formation of β-sheet-rich fibrillar aggregates^42–44^. Such transitions are characterized by a decrease of α-helical content, increase in β-strands content, almost unchanged turn content and increase of unordered structure content^45,46^. In contrast to these cases, presenilin showed a linear relation between α-helical content and β-structure. This behavior represents further evidence for the existence of two different structural states of presenilin. Interestingly, it was observed by *in vitro* studies that such a linear structural transition was a critical step mediating the fibrillogenesis process of Aβ^47^. During the temperature induced aggregation of Aβ (1–40) the conversion of α-helix contributed mainly to the nascent β-strands^47,48^. The ThT and the Congo Red assay showed that the observed structural transition of presenilin leads to signals comparable to BSA fibril formation. The re-cooled protein refolded to some extent, but the refolded structure exhibited a substantial amount of anti-parallel beta-structure, which was virtually absent in the native protein. It appears therefore likely, that the intermolecular interaction involves the formation of anti-parallel beta-structure formed by the thermally induced structural transition. Consequently it has to be concluded that the aggregation stabilizes the high temperature state of PSs. The PS proteins carrying the FAD mutations, which are more susceptible to the transformation than the native protein, are even more likely to adopt the β- structure rich state.

## Methods

### Materials

The expression *E. coli* strains BL21 (DE3) and C43 (DE3) were purchased from Invitrogen and Lucigen respectively. Detergents and Ni-NTA resin used for solubilization and purification were obtained from Cube Biotech. EDTA-free protease inhibitor cocktail was purchased from Roche. A HiLoad 16/60 Superdex 200 pg column was obtained from GE Healthcare. Other reagents were purchased from either Sigma-Aldrich or Merck.

### Plasmids construction

The codon-usage optimized human gene PS2 and PS1 were purchased from Genart and cloned into the pQE2 vector (Qiagen) with an N-terminal hexa-histidine tag. PS1 constructs carrying AD related mutations were generated using the QuikChange Site-Directed Mutagenesis Kit (Agilent). The sequences of obtained plasmids were confirmed by sequencing (see Supplementary Table S2).

### Protein expression and enrichment

Different PSs constructs were expressed and purified by immobilized metal affinity chromatography (IMAC) and SEC according to previously reported methods^20,23,49^. Briefly, the protein was solubilized in high excess of detergent (1% (w/v)) in solubilization buffer (20 mM Tris·HCl, pH 8.0; 10% (v/v) Glycerol; 300 mM NaCl; 1 mM PMSF; 1 tablet protease inhibitor per 50 ml buffer; 1 mM TCEP) over night at 4 °C. Membrane suspensions corresponding to identical amounts of cell pellet were used for purification to compare the yield for detergents.The protein was enriched by Ni-NTA affinity chromatography and eluted by 300 mM imidazole in SEC buffer (20 mM Hepes, pH 8.0; 300 mM NaCl; 10% Glycerol; 1 mM TCEP and either 0.141% (w/v) FC12, 0.0138% (w/v) FC14, 0.00159% (w/v) FC16 or 0.0261% (w/v) DDM)^20^.

Initially, for solubilization a high excess of detergent (1% (w/v)) was used to allow complete solubilization. During purification, this excess was stepwise decreased to obtain finally (SEC) the protein in low excess of detergent with roughly 3 CMC (in pure water) to avoid artifacts in fluorescence measurement due to high scattering and interference in labeling experiments.

### Purification and determination of the apparent molecular weight for presenilin-detergent complexes by SEC

The total amount of the IMAC purified detergent-PSs complexes were concentrated (30 kDa concentrator, Millipore) and filtrated (0.2 µm centrifugation filter, Nanosep) before being subjected to the first SEC. SEC was carried out on a Superdex 200 pg column (GE Healthcare) pre-equilibrated with 2 column volumes of SEC buffer at a flow rate of 0.3 ml/min. For further analysis the non-aggregated material was pooled and the aggregated material (elution volume < 75 ml) discarded (see Supplementary Figure S1). For analysis of the apparent molecular weight the SEC was re-run under the same conditions.

Column calibration was performed under the same experimental condition by a SEC calibration kit (Sigma-Aldrich). The apparent molecular weights of the eluted protein detergent complexes were obtained from the calibration curve (see Supplementary Table S1).

The molecular weights of detergent micelles were determined from SEC elution volumes at 576 nm for lissamine rhodamine B PE labeled micelles by adding the lissamine rhodamine B (Avanti) to final concentration of ~192 nM^20^. The molecular weights of detergent micelle in actual SEC buffer were calculated from the calibration curve (see Supplementary Figure S2). Phosphocholine detergents were reported to form spherical micelles^50^, which allows the estimation of the oligomersiation state of the protein by subtracting the apparent Mw of the empty detergent micelle from the apparent Mw of the protein-detergent complex^51^ (see Supplementary Table S1).

### Fluorescence and CD spectroscopy

Buffer exchanges into CD buffer (10 mM Na_2_PO_4,_ pH 7.4, 150 mM NaF and either 0.141% (w/v) FC12, 0.0138% (w/v) FC14 or 0.00159% (w/v) FC16) was carried out by PD-10 columns (GE Healthcare). Far UV CD spectra from 260 nm to 185 nm with wavelength steps of 1 nm, a bandwidth of 1 nm and an averaging time of 9 s were collected with 1 mm path length cuvettes at protein concentrations of ~3 µM using an Aviv 425 CD spectrometer. For near-UV CD, spectra from 350 nm to 250 nm were recorded with 0.25 nm step size, 1 nm bandwidth, and 30 s averaging time in 1 cm path length cuvettes at concentrations of ~18 µM protein in SEC buffer. All CD spectra were measured at 4 °C except for thermal unfolding experiment. Spectra were corrected for buffer contribution and scaled to obtain mean residue ellipticities (MRE). Experimental reproducibility was accessed by triplicate measurement (see Supplementary Figure S10). The influence of the deconvolution algorithm was monitored by processing the data by CDSSTR^52,53^ (with reference data set SMP180) and BeStSel^54^ (see Supplementary Figure S10).

Tryptophan fluorescence emission spectra (450 to 310 nm with wavelength steps of 1 nm, an excitation wavelength of 295 nm, a bandwidth of 2 nm, a PMT voltage of 900 V and an emission slit width of 2 mm) were recorded using an Aviv 425 CD spectrometer equipped with an fluorescence emission scanning monochromator which allows simultaneous collection of CD and fluorescence data. The fluorescence spectra were normalized for protein concentration and excitation light intensity by the water Raman scattering peak^55^.

Protein thermal unfolding was monitored either by recording CD spectra (260 nm to 190 nm) or monitoring the CD signal at 221 nm and 209 nm with a heating rate of 1 K per min in 2 K increments from 277.15 to 371.15 K. The sample was allowed to equilibrate to the set temperature for 3 min. before measurement.

Based on a two state transition of thermal unfolding, the degree of unfolding was expressed as fraction unfolded (f _U_) using equation (1)^56^.

1 $${f}_{{\rm{U}}}=({{\rm{I}}}_{{\rm{S}}}-{\rm{I}}({\rm{T}}))/({{\rm{I}}}_{{\rm{S}}}-{{\rm{I}}}_{{\rm{E}}}){{\rm{I}}}_{{\rm{S}}},{{\rm{I}}}_{{\rm{E}}}:\mathrm{Intensity}\,{\rm{at}}\,{\rm{start}}\,{\rm{and}}\,{\rm{end}}\,{\rm{temperature}}\,({\rm{T}})\,{\rm{of}}\,{\rm{unfolding}}$$

To obtain T_m_ (melting temperature) or T_t_ (transition temperature), *f*_U_ was plotted as a function of temperature and fitted into the equation (2)^57,58^ using Origin 9.0 (OriginLab).

2 $${f}_{{\rm{U}}}=(({{\rm{f}}}_{u,S}+{\rm{M}}\ast {\rm{T}})+({{\rm{f}}}_{u,E}+{\rm{N}}\ast {\rm{T}})\ast \exp \,({\rm{E}}\ast (1/{{\rm{T}}}_{{\rm{m}}}-1/T)))/(1+\exp ({\rm{E}}\ast (1/{{\rm{T}}}_{{\rm{m}}}-1/{\rm{T}})))$$

f_**u,S**_ and f_**u,E**_ is the unfolded fraction at start and end temperature, M is the slope at low temperature, N is the slope at high temperature, E is ΔH/R, T is the temperature.

### Thermal shift assay

2 μl of 12.5 times concentered protein samples (~30 µM by 10 kDa cut off concentrator) and 2 μl of SYPRO Orange solution (62.5X stock) were added to SEC buffer without detergent to a total volume of 25 μl. The final detergent concentration in the assay will be similar as the original stock assuming that FC14 micelle is also concentrated. The temperature was increased from 278.15 to 368.15 K using a heating rate of 1 K/min in 1 K steps with 5 minutes equilibration time before the measurement. Sample buffer and Milli-Q water were used for controls. SYPRO Orange fluorescence as a function of temperature was recorded by a Biorad MyIQ system (Ex: 485/20X, Em: 530/20X).

### Formation of temperature resistant protein aggregates probed by electrophoresis

Purified PS2-FC14 complex (~3 µM) was unfolded at a heating rate of 1 K/min as in the CD melting experiments. At the respective temperatures an aliquot of 3.5 ug of PS2 was mixed with SDS sample buffer and analyzed by SDS-PAGE and blue-silver staining^59^. This amount of protein showed minimal oligomerization in the SDS-PAGE without pre-heating. The relative amount of each species in the respective lane was obtained densitometrically (image processing by ImageJ^60^). Signal strength of the protein band as a fraction of the total signal of the lane was analyzed as a function of temperature. The data were fitted into equation (2) to obtain T_m_ or T_t_.

### Thoflavin T and Congo Red assays

The formation of amyloid fibrils can be probed *in vitro* by using amyloid-specific dyes Thioflavin T (ThT) and Congo Red (CR)^61^ which bind to ordered protein aggregates rich in beta pleated sheet conformation^62^. ThT undergo an increase in fluorescence upon binding to ordered aggregates which are characterized by cross-β-sheet rich structure. CR exhibits an absorption maximum at ~480 nm because of the π–π * transition of the azo group. Upon binding to amyloid fibrils the intensity of these bands increase and shift to 510–540 nm.

ThT and CR were purchased from Sigma-Aldrich and used without further purification. Dye stock was prepared by dissolving solid powder in PBS buffer (10 mM Na_2_HPO_4_, 1.8 mM KH_2_PO_4_, 137 mM NaCl, 2.7 mM KCl) and was filtered twice through a 0.2 µm syringe filter. Then the stock concentration was determined using an extinction coefficient of 26.6 × 10^3^ cm^−1^ M^−1^ for ThT and 5.53 × 10^4^ cm^−1^ M^−1^ for CR respectively.

Purified PS2 (~13 µM) in SEC buffer was aliquoted into two aliquots. One aliquot was unfolded by incubation at 80 °C for 12 h. The other aliquot, the native protein control was kept frozen until measurement. For the assay both protein stocks were allowed to equilibrate to 24 °C for 2 h.

For the ThT binding fluorescence assay 2 µL of ThT stock were mixed with 100 µL protein stock and 198 µL of SEC buffer to final concentrations of 20.7 µM of ThT and 4.3 µM of protein respectively. These solutions were incubated at the measurement temperature of 24 °C for 15 min before measurement. The ThT fluorescence was excited at 440 nm (5 nm excitation bandwidth) and recorded in the range of 600 nm to 460 nm from a 1 mm path length cuvette.

For the CR binding assay 1 µL of CR stock was mixed with 100 µL protein stock solution and 700 µL of SEC buffer to final concentration of 7 µM of CR and 1.6 µM of protein respectively. The solution was incubated at the measurement temperature of 24 °C for 15 min before measurement. The CR UV absorbance spectra were recorded between 700 nm to 400 nm. The spectra were corrected by baseline subtraction for buffer contribution.

In both assays BSA (15 µM in PBS) was used as a positive control^29^. To probe the structural difference between unfolded and folded protein, CD spectra of the equally prepared samples in PBS buffer were collected for the label free reference materials.

### Detergent critical micelle concentration determination

The critical micelle concentration (cmc) was measured by fluorimetric titration of ANS (8-Anilino-1-naphthalenesulfonic acid, obtained from Sigma-Adrich) with different detergents as described^63^. Briefly, titration was carried out at 4 °C by adding increasing volumes of 1 µl to 20 µl of a detergent stock solution (200 mM FC12, 20 mM FC14, 2 mM FC16, 25 mM DDM) to 50 µM ANS in either SEC or CD buffer (2.8 ml). The emission of ANS was monitored at 490 nm with 370 nm excitation (bandwidth 2 nm). The fluorescence intensity was corrected for dilution. The cmc was obtained from the intersection point of two linear fits describing the curves (see Supplementary Figure S11b).

## Electronic supplementary material

Supplementary Information
