# Supplementary material for: Influence of solubilization and AD-mutations on stability and structure of human presenilins
Source: Sci Rep. 2017 Dec 21;7:17970. doi: 10.1038/s41598-017-18313-x (PMC5740079; doi:10.1038/s41598-017-18313-x)
Supplement: Supplementary file 1 — Supplementary Information [file 41598_2017_18313_MOESM1_ESM.pdf]

Influence of solubilization and AD-mutations on stability and structure of human presenilins

Ge Yang[1, 2], Kun Yu[1, 2], Christina-Symina Kaitatzi[1,3], Abhilasha Singh[1, 2] and Jörg Labahn[1, 2, 4*]

[1] Centre for Structural Systems Biology (CSSB), CSSB-FZJ, Notkestr. 85, 22607 Hamburg, Germany

[2] Institute of Complex Systems-Structural Biochemistry (ICS-6), Forschungszentrum Jülich, Wilhelm-Johnen-Str., 52425 Jülich, Germany

[3] Physics Department, University of Patras, University Campus, 26504 Rio Achaia, Greece

[4] Institut für Physikalische Biologie, Heinrich-Heine-University Düsseldorf, Universitätsstraße 1, 40225 Düsseldorf, Germany

[*] Corresponding author

Corresponding author

Jörg Labahn

Centre for Structural Systems Biology (CSSB), CSSB-FZJ, Notkestr. 85, 22607 Hamburg, Germany
Tel: +49 40 8998-87540
Email: j.labahn@fz-juelich.de

Supplementary data

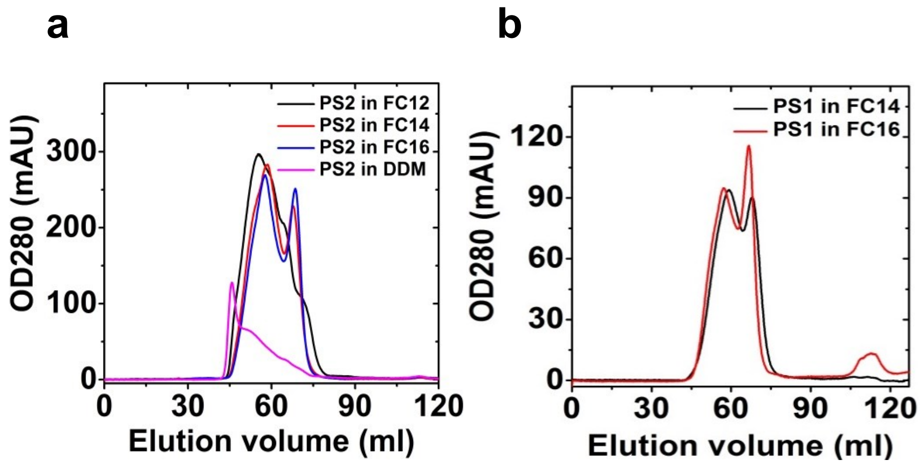

Supplementary Figure S1: SEC elution profiles of purified presenilins in different detergents.

a, First SEC of PS2 after Ni-NTA; b, First SEC of PS1 after Ni-NTA. The low molecular weight state (right peak) was pooled for further analysis. For data on column calibration see Supplementary Figure S2 online.

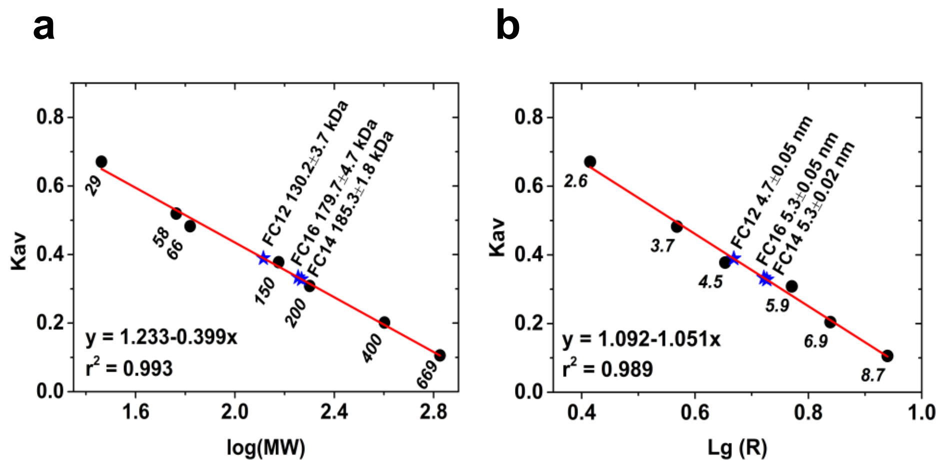

Supplementary Figure S2. Molecular weight (a) and hydrodynamic radius (b) determination of presenilin2-detergent complex.

Superdex 200 pg column was calibrated by a SEC calibration kit. Black points represent the protein standards and blue stars are PS2-detergent complex. The MW (kDa) and hydrodynamic radius (nm) of protein standards are indicated in the respective plot. The calibration applies to data shown in Fig.1, Supplementary Figure S1 and Supplementary Table S1 online.

a

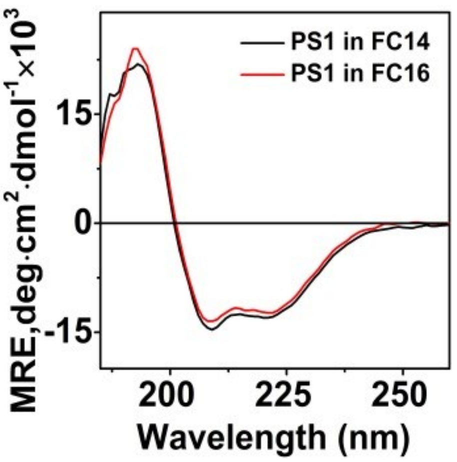

b

Bestsel	Helix		Strand				Turn	Others	NRMSD
Sample	Helix1	Helix2	Anti1	Anti2	Anti3	Para			
PS1 in FC14	21.9	14.5	0.0	1.8	0.8	5.2	13.2	42.6	0.0835
PS1 in FC16	21.4	11.7	0.0	1.7	5.0	3.7	13.3	43.2	0.0901

Supplementary Figure S3: Far UV CD spectra (a) and deconvolution (b) of presenilin1 in fos-choline detergents.
See Methods for experimental conditions. The deconvolution was performed by BeStSel [1]. Structure content shown in percent.

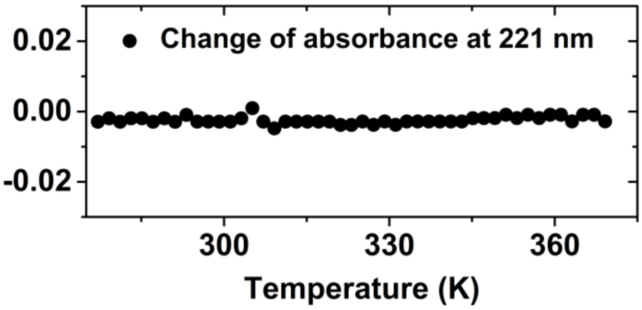

Supplementary Figure S4. Change of absorbance at 221 nm during thermal unfolding. Precipitation control for thermal unfolding of PS2 in FC14 shown Fig.4. Change of absorbance at 221 nm was calculated using the following formula: ΔAbs = Gain * log (V/Vo); where Abs is absorbance, gain is the PMT gain (7), V and Vo are the subsequent dynode voltages.

a

Bestsel	Helix		Strand				Turn	Others	NRMSD
Temp (K)	Helix1	Helix2	Anti1	Anti2	Anti3	Para			
277.15	28.99	15.91	0	1.8	0	2.04	13.58	37.68	0.003
285.15	28.23	15.33	0	1.63	0.43	1.8	12.8	39.8	0.0038
293.15	26.65	15.36	0	2.14	0.23	2.62	13.29	39.71	0.0037
301.15	27.84	15.05	0	2.49	1.5	2.04	13.25	37.81	0.0043
309.15	24.46	15.29	0	1.58	0.49	2.93	12.9	42.35	0.0057
317.15	23.47	14.18	0	3.41	0	3.48	12.92	42.54	0.0056
325.15	21.06	12.12	0	5.16	0.28	5.8	13.31	42.28	0.0055
333.15	18.15	10.33	0	7.9	0.45	7.94	12.68	42.55	0.0058
341.15	16.39	8.11	0	8.74	2.18	9.37	12.57	42.64	0.0056
349.15	11.99	6.98	1.48	11.79	3.72	11.07	10.95	42.03	0.0074
357.15	10.87	6.31	0.16	11.16	2.4	12.46	12.05	44.59	0.0062
365.15	10.42	5.77	0.21	10.48	2.18	13.2	11.61	46.14	0.0071
371.15	11.59	6.64	0.1	11.64	0.87	11.99	12.16	45.00	0.0066

b

CDSSTR Temperature (K)	Helix	Strand	Turns	Unordered	NRMSD
277.15	52.0	8.30	12.0	26.3	0.009
285.15	52.0	9.67	13.0	26.0	0.113
293.15	51.7	9.67	13.3	25.7	0.012
301.15	50.7	9.67	13.0	26.7	0.013
309.15	50.3	8.67	14.3	26.7	0.016
317.15	48.3	11.3	14.0	27.0	0.018
325.15	43.3	14.3	15.0	27.0	0.016
333.15	36.7	19.7	15.3	28.0	0.018
341.15	33.0	23.3	16.3	28.3	0.021
349.15	23.0	25.7	16.3	29.0	0.019
357.15	29.3	24.7	17.0	29.0	0.021
365.15	27.0	27.0	17.3	28.7	0.020
371.15	26.0	27.3	17.3	29.7	0.021

Supplementary Fig S5: Comparison of secondary structure deconvolutions from different algorithms.

Secondary structure components of PS2 in FC14 during thermal unfolding. For presentation of analysis see Fig.4. a, Deconvolution of Secondary structure components by BeStSeL; b, Deconvolution of Secondary structure components by CDSSTR. The values represent mean ± standard deviation between the deconvolutions obtained with different reference data sets. NRMSD (normalized root mean square deviation) indicates the best fit between the calculated and experimental CD spectra. Structure content shown in percent.

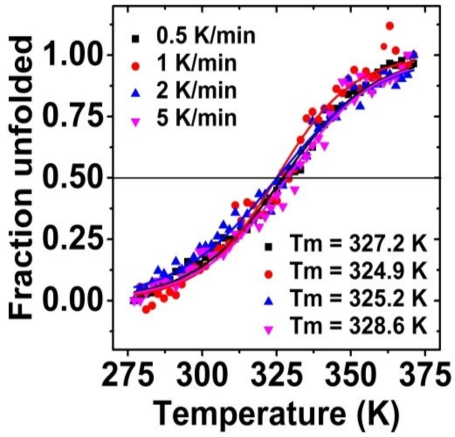

Supplementary Figure S6. Effect of heating rate on the thermal unfolding of PS2 in FC14.

All transition temperatures fall into the range of 324.9 to 328.6 K (mean Tm 326.5 ± 2.1) for variation of the heating rate (step size of 2 degree) from 0.5 to 5 K per min.

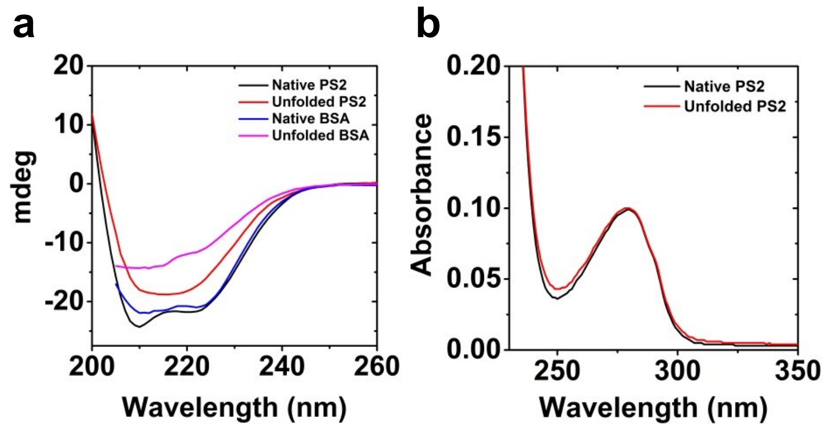

Supplementary Figure S7: Spectroscopic controls for the fibrils assay.

Far UV CD (a) and absorption (b) spectra of folded and unfolded protein. See methods for experimental parameters.

BeStSel	Helix		Strand				Turn	Others	NRMSD
PS2 in FC14	Helix1	Helix2	Anti1	Anti2	Anti3	Para			
277 K	28.74	16.38	0	0.63	0	2.45	12.8	38.99	0.0035
371 K	9.82	4.61	0.17	11.88	3.48	13.33	10.89	45.82	0.0085
Back to 277 K	14.74	7.21	3.63	12.44	6.78	6.41	10.47	38.32	0.003

Supplementary Figure S8: Irreversibility of thermal unfolding of PS2.

Processing of data [2] by BeStSel. CD spectra from PS2 in FC14 (condition see Methods) collected from sample heated from 4 to 98 °C by 2 °C steps with re-cooling to 4 °C. Spectra were deposited in Protein Circular Dichroism Data Bank with PCDDBid: CD0005962000 (4 °C), CD0005963000 (98°C) and CD0005964000 (back to 4 °C). Structure content shown in percent.

a

BeStSel	Helix		Strand				Turn	Others	NRMSD
PS1 in FC16	Helix1	Helix2	Anti1	Anti2	Anti3	Para			
PS1	22.88	12.56	3.24	6.77	7.63	0	12.68	34.25	0.0034
PS1D257A/D385A	23.34	14.02	1.44	4.78	1.79	0.74	14.2	39.69	0.003
PS1L226F	23.08	12.75	2.26	6.74	6.94	0.39	13.07	34.77	0.0027
PS1L424V	21.64	13.18	3.43	7	6.61	0	13.32	34.83	0.0036
PS1M292D	22.01	12.69	2.67	5.78	6.32	1.45	12.47	36.59	0.0032
PS1ΔE9	22.07	12.48	3.33	7.46	7.84	0	12.99	33.83	0.0034

b

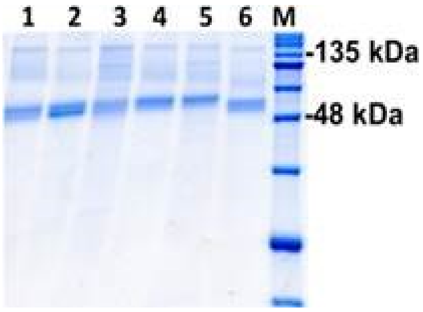

c

Protein	T_m (K)	Stability change (kcal/mol)
PS1	342.5±0.5	0
PS1D257A/D385A	338.9±0.4	n.d.
PS1L226F	335.2±0.5	-2.11
PS1L424V	336.3±0.6	-1.33
PS1M292D	334.7±0.4	n.d.
PS1ΔE9	337.6±0.4	n.d.

Supplementary Figure S9: PS1 and mutants in FC16.

a, Helical content of PS1 AD mutants; b, 12 % SDS-PAGE of ~2 µg of FC16 purified PS1 AD mutants; Lane 1: PS1WT (wild type); lane 2: PS1D257A/D385A; lane 3: PS1L266F; lane 4: PS1L424V; lane 5: PS1M292D; lane 6: PS1ΔE9; M: Prestained Protein Marker (Jena bioscience); c, Melting temperatures obtained from fraction unfolded (see Fig.8c); stabilities upon point mutation were predicted based on the structure of PS1 wild type (5fn2:B) using I-Mutant 2.0 [3]. Stability changes were calculated by ΔG mutant - ΔG wild type. n.d.: structures unknown. Structure content shown in percent.

a

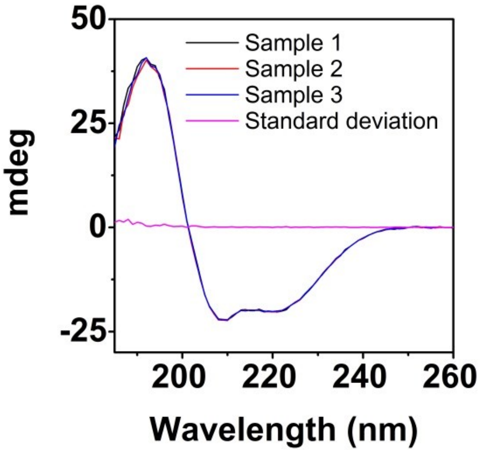

b

CDSSTR	Helix		Strand		Turns	Unordered	NRMSD
PS2 in FC14	Helix R	Helix D	Strand R	Strand D			
1	33	17	7	5	11	29	0.014
2	32	18	6	5	11	29	0.013
3	34	17	6	4	11	28	0.013

c

BeStSel	Helix		Strand				Turn	Others	
PS2 in FC14	Helix1	Helix2	Anti1	Anti2	Anti3	Para			NRMSD
1	29.91	15.64	0.98	2.66	1.86	1.43	13.03	34.5	0.0029
2	28.96	16.52	0	1.13	0.61	2.01	13.44	37.32	0.003
3	29.46	15.94	0	2.21	1.12	1.96	13.75	35.56	0.0028

Supplementary Figure S10: Reproducibility of experimental CD-data and data deconvolution.
a, Far UV CD spectra from 3 PS2 samples in FC14 and the wavelength dependence standard deviation; b, deconvolution of far UV CD spectra from a into secondary structure component by the algorithm CDSSTR [4,5] (with reference data SMP 180); c, deconvolution of far UV CD spectra from a into secondary structure component by the algorithm Bestsel [1]. See methods for conditions. Structure content shown in percent.

Protein detergent complex	Elution volume (ml)	Mw of Protein detergent complex (kDa)	Micelle size by SEC calibration (kDa)[a]	Estimated oligomerization state[b]
**PS2 in FC12**	72.62	126	31.10	1.9
**PS2 in FC14**	67.78	187	51.53	2.7
**PS2 in FC16**	68.62	175	68.55	2.1
**PS2 in DDM**	65.36	228	56.36	3.4
**PS1 in FC14**	67.89	185	51.11	2.4
**PS1 in FC16**	66.71	204	68.55	2.5
**PS1D257A/D385A in FC16**	66.55	207	68.55	2.5
**PS1L226F in FC16**	61.43	314	68.55	4.5
**PS1L424V in FC16**	66.55	207	68.55	2.5
**PS1M292D in FC16**	65.71	221	68.55	2.8
**PS1ΔE9 in FC16**	66.03	216	68.55	2.7

Supplementary Table S1: Apparent molecular of PS detergent complex determined by SEC.
[a] Detergent micelle was stained with lissamine rhodamine B PE and Mw was determined by SEC calibration. [b] The oligomerization state was estimated after correction for the Mw of the detergent micelle (See Methods). For data on column calibration see Supplementary Figure S2.

**Protein**	**Sequence**
**HisPS1 (Mw = 54831 Da)**	MGSSHHHHHHSSGLVPRGSHMTELPAPLSYFQNAQMSEDNHLSNTVRSQNDNRERQE<br>HNDRRSLGHPEPLSNGRPQGNSRQVVEQDEEEDEELTLKYGAKHVIMLFVPVTLCMVVV<br>VATIKSVSFYTRKDGQLIYTPFTEDTETVGQRALHSILNAAIMISVIVVMTILLVVLYKYRCYK<br>VIHAWLIISSLLLLFFFSFIYLGEVFKTYNVAVDYITVALLIWNFGVVGMISIHWKGPLRLQQA<br>YLIMISALMALVFIKYLPEWTAWLILAVISVYDLVAVLCPKGPLRMLVETAQERNETLFPALI<br>YSSTMVWLVNMAEGDPEAQRRVSKNSKYNAESTERESQDTVAENDDGGFSEEWEAQR<br>DSHLGPHRSTPESRAAVQELSSSILAGEDPEERGVKLGLGDFIFYSVLVGKASATASGDW<br>NTTIACFVAILIGLCLTLLLLAIFKKALPALPISITFGLVFYFATDYLVQPFMDQLAFHQFYI
**HisPS2 (Mw = 52293 Da)**	MKHHHHHHLTFMASDSEEEVCDERTSLMSAESPTPRSCQEGRQGPEDGENTAQWRSQ<br>ENEEDGEEDPDRYVCSGVPGRPPGLEEELTLKYGAKHVIMLFVPVTLCMIVVVATIKSVR<br>FYTEKNGQLIYTTFTEDTPSVGQRLLNSVLNTLIMISVIVVMTIFLVVLYKYRCYKFIHGWLI<br>MSSLMLLFLFTYIYLGEVLKTYNVAMDYPTLLLTVWNFGAVGMVCIHWKGPLVLQQAYLI<br>MISALMALVFIKYLPEWSAWVILGAISVYDLVAVLCPKGPLRMLVETAQERNEPIFPALIYS<br>SAMVWTVGMAKLDPSSQGALQLPYDPEMEEDSYDSFGEPSYPEVFEPPLTGYPGEELE<br>EEEERGVKLGLGDFIFYSVLVGKAAATGSGDWNTTLACFVAILIGLCLTLLLLAVFKKALPA<br>LPISITFGLIFYFSTDNLVRPFMDTLASHQLYISAWSHPQFEK

Supplementary Table S2: Molecular weights and sequences.

a

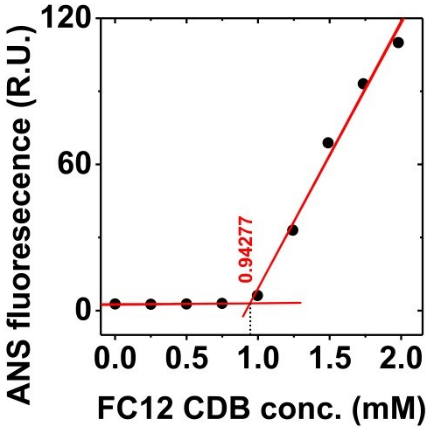

b

Detergents	cmc Water (mM)	cmc SEC buffer (mM)	cmc CD buffer (mM)	Concentration used for SEC and CD (mM)
FC12	1.5	0.86	0.94	4.0
FC14	0.12	0.043	0.069	0.36
FC16	0.013	0.0041	0.0060	0.039
DDM	0.17	0.090	0.10	0.51

Supplementary Figure S11: Critical micelle concentrations determination by ANS titration. a, Representative example of cmc determination; b, List of cmc values of different detergents; Cmc for detergents in SEC buffer (20 mM Hepes, pH 8.0; 300 mM NaCl; 10 % Glycerol; 1 mM TCEP) and CD buffer (10 mM Na_2PO_4, pH 7.4, 150 mM NaF) used in this study were determined by fluorimetric titration. See Methods for experimental conditions. CMC values in water as provided by Anatrace.

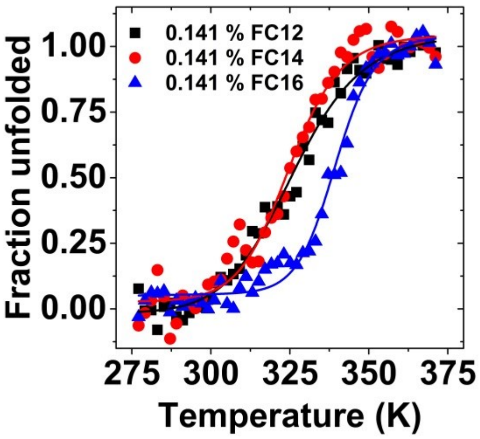

Supplementary Figure S12: Thermal unfolding of PS2 control experiment in constant concentration (w/v) of detergents. Two state transitions analysis yielded transition temperatures of ~ 324 and ~339 K respectively for FC14 and FC16. Increasing the detergent concentrations to 0.141% (w/v) did not alter the transition temperatures (compare Figure 3a).

Supplementary reference

1 Micsonai, A. *et al.* Accurate secondary structure prediction and fold recognition for circular dichroism spectroscopy. *Proc Natl Acad Sci U S A* **112**, E3095-3103, doi:10.1073/pnas.1500851112 (2015).

2 Yang, G., Yu, K., J., K. & Labahn, J. Expression, purification and preliminary characterization of human presenilin-2. *Process Biochemistry* **in press**, doi:http://dx.doi.org/10.1016/j.procbio.2017.09.012 (2017).

3 Capriotti, E., Fariselli, P. & Casadio, R. I-Mutant2.0: predicting stability changes upon mutation from the protein sequence or structure. *Nucleic Acids Res* **33**, W306-310, doi:10.1093/nar/gki375 (2005).

4 Whitmore, L. & Wallace, B. A. Protein secondary structure analyses from circular dichroism spectroscopy: methods and reference databases. *Biopolymers* **89**, 392-400, doi:10.1002/bip.20853 (2008).

5 Whitmore, L. & Wallace, B. A. DICHROWEB, an online server for protein secondary structure analyses from circular dichroism spectroscopic data. *Nucleic Acids Res* **32**, W668-673, doi:10.1093/nar/gkh371 (2004).
